# Academy of Stomatology

**Published:** 1905-08

**Authors:** 

**Affiliations:** Academy of Stomatology


### ACADEMY OF STOMATOLOGY.

A REGULAR meeting of the Academy of Stomatology, of Philadelphia, was held at its rooms, 1731 Chestnut Street, on the evening of March 28, 1905, the President Dr. I. N. Broomell, in the chair. A paper entitled "The Rationale of the Porcelain Inlay" was read by Dr. J. Q. Byram, of Indianapolis, Ind.

(For Dr. Byram's paper, see page 497.)

### DISCUSSION.

*Dr. Joseph Head.*—In talking with Dr. Byram this afternoon he told me that he would consider it a personal favor if, as a scientific man who has worked from a scientific basis, I would adversely criticise his paper, and felt that he would rather have the truth than compliments. Any one who has listened to this paper and seen this array of plates knows that, while the work may be improved upon in the future, it is a distinct advance in dentistry. I feel that we should be very glad that Dr. Byram has been with us this evening.

The essayist spoke of the necessity of making a filling so that it will be held in place, not so much by the cement, as by stepping in the cavity, and that the cement will act merely as a stayer of the filling. I have had that belief for a long time, but in the light of some experiments which I have been carrying on since last August, I still believe that we do not at present thoroughly understand the exact value of cement with reference to a thin line and a thick line. We do not understand whether a thin line is stronger than a coarse line, or whether the cement will act as a glue or as a dowel. In my experiments I put the ordinary Harvard cement under a pressure of eight pounds to ¼-inch square between flat glass plates, and found that the thinnest cement line obtainable was 0.0003 of an inch. I made a long series of experiments, which will be published in the *Dental Cosmos.* For the sake of bringing out the point of the paper, I will give the ultimate tests, to show the adhesive strength of inlays. I drilled into ivory a number of cavities of the standard size of one-eighth inch in diameter and three-sixteenths of an inch in depth, and then made ten porcelain fillings to fit the cavities. A plunger could be inserted from underneath. These fillings were cemented in under ordinary thumb-pressure of about four pounds and pressure continued for about a minute. In testing the amount of force required to pull out the fillings, a spring balance was used coated with carbon, and in that way accurate readings were made. Every one of the glazed fillings came out with a pressure of about six ounces. The same fillings, etched and cemented under the same conditions, were removed at an average of about two pounds. I undercut the ivory, and found the pressure went up to about sixteen pounds for the Harvard, and twelve pounds for the Ames. I then undercut the fillings deeply, and set them again, and found that they came out under twenty-five pounds pressure for the Harvard cement, and nineteen pounds for the Ames. My conclusion was that inlays should always be put in so that there are undercuts in the fillings, undercuts in the dentine, and that the porcelain should also be etched.

The color problem is a difficult one. I feel that if we get a good color, a color of cement that approximates the color of the dentine, perhaps a little lighter, in order to overcome the shadow cast by the layer between the light and the porcelain, we will secure a color about as good as it is possible to get.

The filling that Dr. Byram put in to-day in his clinic was a beautiful match, and I am delighted that it was made in layers.

But when I turned the patient around so that the light struck the tooth, at first it looked something like "blue mud." I think the best we can do, so far, is to put in a porcelain filling that from the general point of view from which it is ordinarily seen will look well.

In reference to the use of pyrometers in the baking of porcelain, I think we have to know how to bake porcelain even with this instrument. The time may vary, and this is the important element. While the pyrometers may be a help, the principle is much the same as in studying Latin composition,—the only way to study it is to study it, and the only way to bake porcelain is just to bake it. The mixing of cement liquids in certain proportions to the powder is fine in theory; the only trouble is that in warm weather the liquid will deliquesce and set in varying lengths of time.

*Dr. W. A. Capon.*—I also would like to thank Dr. Byram for his practical and concise paper, which must be of advantage to all and of particular interest to many. Of course, I do not believe in everything that has been said. I have not made the experiments outside of the mouth which he has, but I have done a great deal of work within the mouth. I think the plates he has shown us are very beautiful, and some of them very practical. All of them may be practical, but they are not exactly my ideal. It is much the same as in the filling of root-canals; different materials are used, and with apparently the best results. The preparation of cavities in the early days did not bother us much, and yet these fillings are still in the mouth, therefore the cement must have had much to do with their retention. Now that more attention is paid to the preparation of cavities, we should have better results, and I believe we shall have. I think the diagrams Nos. 1, 2, 3, and 4 are all practical. No. 6 I object to on account of the little piece of porcelain extended on the incisal edge. That will break off after a little wear. I had an illustration of this in a clinical case a couple of years ago. I do not see the advantage of such angles as shown in Fig. 7, and think that the graceful lines belonging to porcelain fillings are destroyed by such angles. In preparing such a cavity I do away with them entirely. Fig. 8 is all right, but it is an exceptional cavity.

I ceased using the pins seen in Fig. 9 ten years ago, and substitute the loop of platinum wire, because the ends are kept in position more easily, and they help to retain the cement.

In the making of the matrix, I agree with the essayist that there is only one way to do it, which is to work on the tooth proper. I never seem to get as nice adaptation as on natural enamel, although we hear of some men doing beautiful work by the swaging process.

In reference to the porcelain basal shades, I think it is a splendid idea to follow out the shade of the tooth. I do that and finish with enamels, but I, with others, know the disadvantage of an opaque substance like cement as an attachment.

I do not think that white cements give the best results in point of strength. The cement should be shaded to suit the tooth as nearly as possible. The cavity preparation has a great deal to do with the retention of the inlay and the ultimate results, and almost any good cement will hold a well-made and placed inlay.

*Dr. J. H. Gaskill.*—I do not see why I should be called upon to discuss this paper, because I am not a specialist nor an enthusiast on the subject of porcelains, but I am interested in some of the results which I have seen, and do some porcelain work. I have been much interested in Dr. Byram's paper, and I think he has taught us a great deal. In his clinic this afternoon he showed by his technic and manipulation his thorough mastery of the art of porcelain inlay work. I could not help feeling, however, that he carried his cutting of tooth-structure to extremes. He made use of the expression, "I cut until I get what I want." Is he justified in getting what he wants? From the cavity which he filled this afternoon he took a gold filling. A comparatively small portion of the tooth was gone from the labial side, but before he was through with it the third of the tooth was sacrificed. His excuse was that the enamel was checked. He did not take into consideration the support which the cement gives the porcelain inlay. I think we see many cases in which, with the lingual surface gone and the labial surface intact, we get æsthetically a better piece of work. You may put an inlay in place, and in certain lights it matches beautifully; in other lights it is very dark. This may be extreme, but it illustrates the condition which we have to meet.

As Dr. Byram stood in a strong light this afternoon and I noticed his teeth, I thought for the moment that he was negligent of cleansing his mouth. I looked again and found that his teeth were filled with porcelain inlays. They were beautifully put in, and the color in most of them was beautiful, but as he stood in certain lights there was a dark line where the cement had washed out, giving the appearance of a carelessly kept mouth. In filling with gold underneath, as a good and experienced operator should do, a great deal of gold can be kept out of sight, and a better piece of work will be the result. The cement problem is the one vital point in inlay work. I agree with Dr. Byram as against Dr. Capon in the results of the use of white cement. Dr. Head uses the Harvard cement, which they call white, but it takes a bluish tinge. The reflection against the whitish cement gives better results.

In the preparation of cavities Dr. Byram has, I think, developed a system which is wonderful as to the shaping of the edges so that there is a mechanical retention of the inlay, and the cement becomes merely a necessary adjunct, because the force of stress is in such a direction that the pressure locks the inlay in position.

I wish to thank Dr. Byram for his paper and for the clinic.

*Dr. G. F. Root.*—Dr. Byram's technic is certainly fine, but I believe one has to have the ideal cases in which to use ideal technic. As a rule, each case presents problems that have to be solved, and we cannot always use the methods which our theory teaches us are the best.

I have seen some teeth filled with porcelain which were blue at the gingival border.

*Dr. Chas. R. Turner.*—I was impressed with one feature of Dr. Byram's paper, which indicated that he was dealing with the practical rather than with the ideal. As I looked at the cavities and heard his description of the technic of filling them, it occurred to me that they might be cavities in any teeth, and not necessarily cavities that present themselves under ideal conditions. In order to get them into the exact shape in which they are presented, what has been termed "sacrifice of tooth-structure" is necessary, but, inasmuch as the ultimate success of the operation is the point in view, it is justifiable; in other words it is a sacrifice of tooth-structure for the preservation of the balance of the tooth.

I was also interested in the very scientific attitude taken by the essayist upon the question of color. The backing up of the inlay with a white cement struck me as a good idea.

In conclusion, I wish to compliment the essayist upon the great detail with which he has worked out his technic, and to thank him personally for the presentation in a very succinct and comprehensive form of the general problem of porcelain inlay.

*Dr. Roberts.*—There has been considerable discussion about the ideal cavity and the inability to find cavities that could be so prepared as to be exactly like the drawings which the essayist has presented. My idea of successful inlay work is for a man to make a cavity ideal and representative without necessarily following any of the exact lines pointed out by the essayist. If he finds that he has a small cavity and one that will not show, he certainly will not sacrifice a great amount of tooth-structure to bring about a cavity similar to that of Fig. 2. He will prepare a cavity so as to save all the tooth-structure he can, and yet get it on ideal lines. If it be a small cavity, he will use gold, while the successful inlay worker will take any cavity and so prepare it that it will in a measure be self-retentive to the filling, the cement simply keeping the porcelain in place, and he does not have to follow any of the ideal shapes here presented. He makes his ideals as he progresses.

I was much interested in the paper, and congratulate the essayist upon the able manner in which it was presented.

*Dr. Byram* (closing).—I first want to thank you for the kind reception given me. As I remarked to my friend, Dr. Head, if there is any one thing I dislike, it is to read a paper and then have every one "agree with the essayist." I take it as a favor to have criticism.

I take it that we are all striving for knowledge on the subject of inlays. While I am here as your guest, and an enthusiastic inlay worker, I want it distinctly understood that I am but a novice. The more I study the subject, the more I find I do not know. I have received many good suggestions since coming to your city which will be of value to me after my return.

I congratulate Dr. Head upon the thorough manner in which he has been testing cements. I believe that he is doing one of the best works in the subject that could be done. I only wish I could join him in this line of experimental work.

There seems to be a difference of opinion as to what I mean by constructing inlays in layers. Dr. Head does the work as I

do in many cases, with a few modifications. I first select my basal color, which I hope will represent as nearly as possible the color of the dentine. Then I select the color of the enamel in the different locations. If it is brown or yellow in the gingival, then I select enamel of a corresponding color. For the incisal I usually select enamel of a bluish hue. After the colors have been biscuited, I apply a uniform color over the entire mass and fuse.

In writing a paper of this kind it must be taken into consideration that there are many points of interest to the beginners in this work which are not of interest to those who are familiar with the details of porcelain work. I do not do everything just as I have stated in this paper. Those experienced in teaching know that we must teach beginners to do that which they can do when they commence, not what they will do in three or four years. I do not always apply my color as I have said. I can hardly see how any one can construct an inlay extending from the gingival line to the incisal edge and use but one colored porcelain. I have yet to see one tooth in which there are not two and usually three shades of color. By selecting porcelains that more nearly approach the color of the tooth in their respective locations I can do better work. If that is what is called constructing in layers, then I am an advocate of the layer method.

In regard to the pyrometer, I may say that I have never had anything in my porcelain work that has given me the pleasure my pyrometer has, and if I know anything about the fusing of porcelain it has been learned since using this instrument. The pyrometer has assisted me in obtaining accurate results, and it has been instrumental in teaching me how to fuse porcelain. To obtain accurate results, you must fuse under exactly the same conditions.

I find that I get better results in mixing cements by weighing the parts. Changes may have to be made according to the weather.

In reply to Dr. Capon, I would say that pioneers in any line of work have done good service. When I see some of the gold fillings that were inserted forty years ago, I am ashamed of my work. When we think of how these men labored preparing their cavities by hand, and taking hours at it, and when we remember how they mastered the technic of inserting these fillings by hand, I say it makes me feel ashamed when I look at many of our gold fillings. Those who began inlay work fifteen or twenty years ago are in the same position. They did not have the modern improvements, porcelains, furnaces, etc. They had to become skilful at once. Those entering this work to-day are not required to go through such experimental work as were Dr. Capon and Dr. Head, and that is the reason we are not so skilful as those who started years ago.

In reference to the thickness of the incisal edge, there are certain cases in which the edges are thick enough to prepare the cavities according to Fig. 6. It is impossible to use these reverse curves without having more space than with some of the other forms of cavity preparation.

In reply to Dr. Gaskill, I will say that in the preparation of a cavity every man must have his ideal, and while I may not carry out in detail my ideal, I try to work to that as nearly as possible. My ideal is to have the cavity as nearly self-retentive as possible. In regard to cutting, I will say that I do cut. Some ask if the patient will stand it? My reply is, I am the dentist, and if they want me to insert inlays, they must submit to my method of operation. We were taught not to leave frail walls for gold fillings, and I would not have you believe that you may leave frail walls for inlays and expect them to give the best service.

In the case of this afternoon I did not make the excuse that the enamel was checked. The cavity was not prepared in an ideal manner, nor under ideal conditions. It was half-past two when I commenced, and it was three o'clock when we secured an engine, and to have prepared the cavity in an ideal manner and constructed the inlay would have taken until seven o'clock. The case was that of a student, and if the filling comes out it can easily be replaced.

Dr. Capon thinks the matrix lining is good because it is white. I do not understand why he would not use a white cement for the same reason that he uses the matrix lining, because the matrix lining is only next to the cement, and he has the enamel over all. If the white cement is used, there is a white film between the inlay and the tooth. Dr. Capon also feels that the white cement or the yellow has not the strength of the darker ones. I would like to ask Dr. Head if he found this to be so in his tests?

*Dr. Head* (answering Dr. Byram).—I was much surprised to hear Dr. Capon say what he did. Although I have not gone into the tests as much as I hope to, yet it seemed to me that, since the dark cements are made so by means of foreign pigments, and the light yellow cement is pure oxide of zinc as it is brought from the crucible, the uncolored cement ought to be stronger than, or at least quite as strong as, the dark.

*Dr. Byram.*—So, I say again that I believe the white cement, or as white as we can get it, is the best cement to use in setting inlays, because we have a cement that has the least power of absorption and the greatest power of reflection of our light rays. For the first few weeks there will be a white line, but in a few weeks it will be just about as brown as if you had used the yellow cement. I will have to offer a word of apology about the inlays in my mouth.

*Dr. Gaskill.*—I was not questioning the workmanship of those inlays, because they are beautiful and the color is good; but simply meant to say that in certain lights the reflection gives the bluish or opaque appearance which you are bound to get in certain lights.

*Dr. Byram.*—I have been experimenting with this matrix lining in my mouth, and, like Dr. Capon, I find I shall have to learn to apply my colors again. A busy man, however, has not time to have inlays made for his mouth more than once. I wish to say that I have some inlays of which I am very proud, put in under my direction by one of my associates. They are the first inlays he ever constructed, except two by the Jenkins system made by him last summer.

*Dr. Head.*—When I spoke of "blue mud," I did not mean to intimate that the filling was not all right, but simply that it followed the law of all these fillings when the light strikes them.

*Dr. Byram.*—I think Dr. Head's remarks were clear. In my paper I tried to make it plain that when the light shines in certain directions against the fillings we always have that change in color.

I would like to meet those of you who are particularly interested in inlay work to exhibit some inlay burs. I am to meet a representative of the S. S. White Dental Company to-morrow, and your endorsement will assist me in having them place the burs on the market.

I would like to say in conclusion that I appreciate the kindness and honor that have been bestowed upon me by the members of this Academy in inviting me here. Coming as I do from a distant State, practically unknown to you, I assure you that I feel very grateful to you.

Otto E. Inglis,
*Editor Academy of Stomatology.*

